# Supplementary material for: Targeting circDGKD Intercepts TKI’s Effects on Up-Regulation of Estrogen Receptor β and Vasculogenic Mimicry in Renal Cell Carcinoma
Source: Cancers (Basel). 2022 Mar 23;14(7):1639. doi: 10.3390/cancers14071639 (PMC8996923; doi:10.3390/cancers14071639)

Supplementary Materials

S1

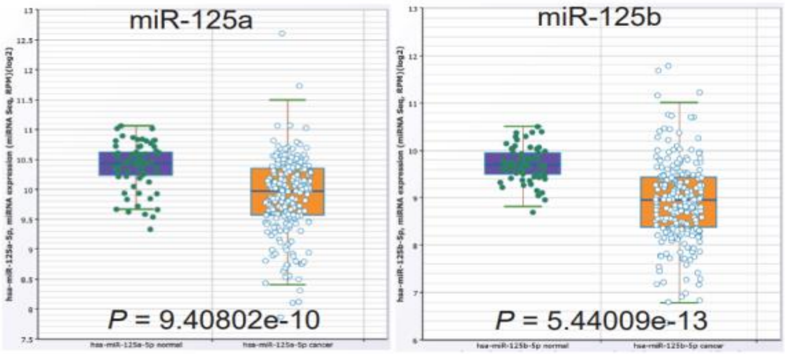

Figure S1. Clinical data from Starbase confirmed the down-regulation of miR-125a-5p and miR-125b-5p in ccRCC patients.

S2

name	mirAccession	geneName	targetSites	bioComplex	clipReadNum
hsa-miR-125b-5p	MIMAT0000423	DGKD_hsa_circ_001411	1	2	132
hsa-miR-125b-5p	MIMAT0000423	BPTF_hsa_circ_001730	1	1	1
hsa-miR-125b-5p	MIMAT0000423	DHPS_hsa_circ_000261	1	2	12
hsa-miR-125b-5p	MIMAT0000423	ERBB2IP_hsa_circ_001427	1	4	75
hsa-miR-125b-5p	MIMAT0000423	APOBEC3C_hsa_circ_001120	1	1	11
hsa-miR-125b-5p	MIMAT0000423	RNF38_hsa_circ_001613	1	1	6
hsa-miR-125b-5p	MIMAT0000423	ANKIB1_hsa_circ_001193	1	3	23
hsa-miR-125b-5p	MIMAT0000423	ADPGK_hsa_circ_000607	1	3	13
hsa-miR-125b-5p	MIMAT0000423	BAZ1B_hsa_circ_001192	1	4	22
hsa-miR-125b-5p	MIMAT0000423	MLL3_hsa_circ_000753	1	2	2454
hsa-miR-125b-5p	MIMAT0000423	ELF2_hsa_circ_001518	1	1	446

Figure S2. Starbase analysis result of the top 10 potential circRNA candidates that might sponge miR-125-5p. Due to information mismatch between Starbase and Circbase for MLL3, the original 11th ELF2 was chosen to be among the top 10 candidates.

S3

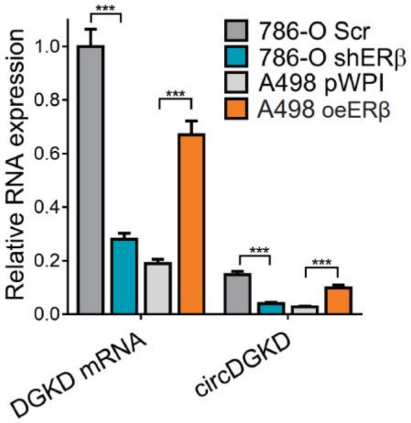

Figure S3. qPCR of the expressions of DGKD mRNA and circDGKD after knocking down ERβ in 786-O cells (shERβ vs. Scramble control), or the overexpressing ERβ in A498 cells (oeERβ vs. pWPI vector control). Data are presented as mean ± SEM. *** $p < 0.001$.

Uncropped Western Blots

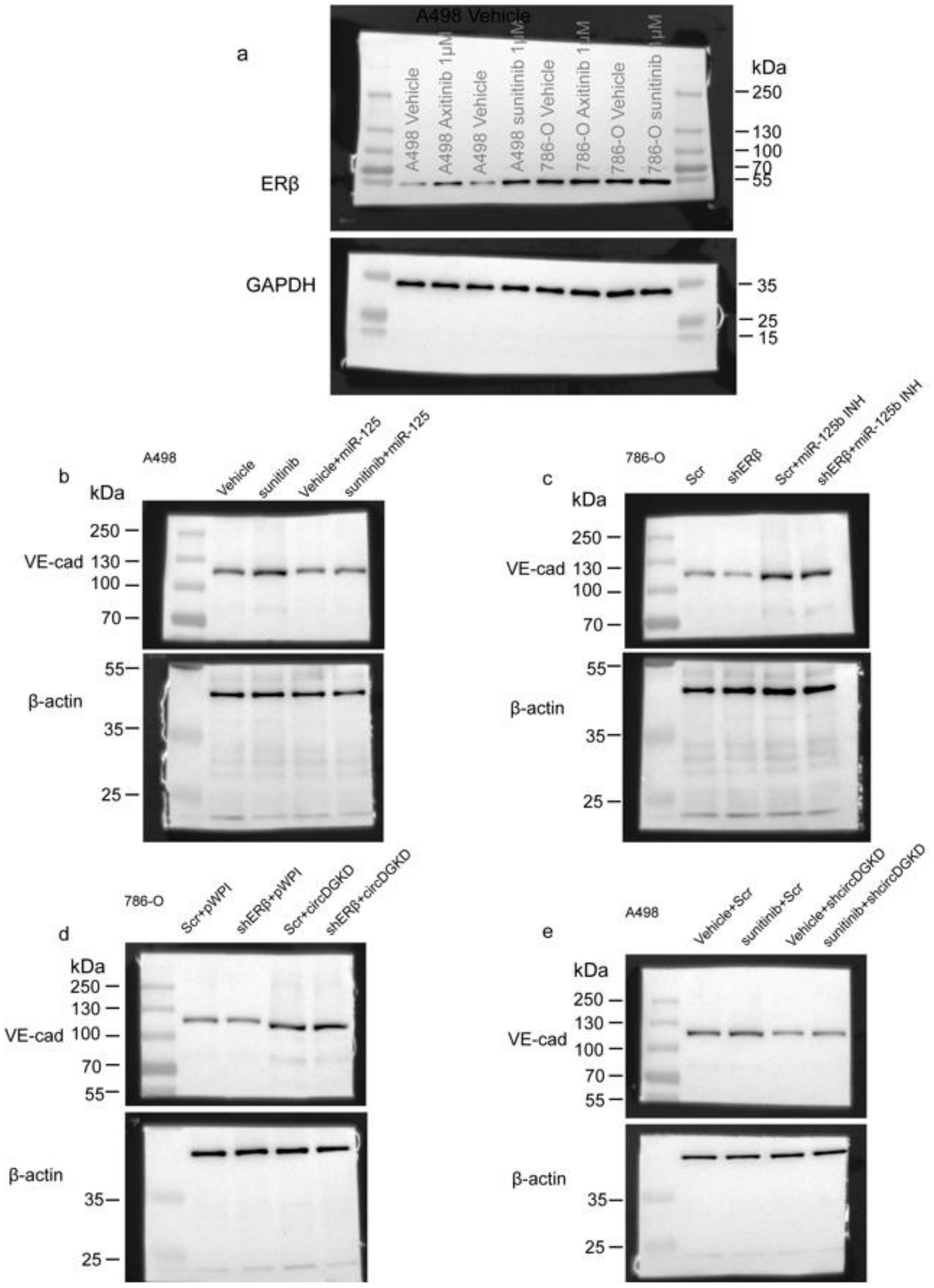

Supplement: Supplementary file 1 [file cancers-14-01639-s001.zip › cancers-1587165-supplementary/Cancers-1587165-Suppl Materials.pdf]
